# Cellular tagging as a neural network mechanism for behavioural tagging

**DOI:** 10.1038/ncomms12319

**Published:** 2016-08-01

**Authors:** Masanori Nomoto, Noriaki Ohkawa, Hirofumi Nishizono, Jun Yokose, Akinobu Suzuki, Mina Matsuo, Shuhei Tsujimura, Yukari Takahashi, Masashi Nagase, Ayako M. Watabe, Fusao Kato, Kaoru Inokuchi

**Affiliations:** 1 Department of Biochemistry, Graduate School of Medicine and Pharmaceutical Sciences, University of Toyama, 2630 Sugitani, Toyama 930-0194, Japan; 2 CREST, JST, University of Toyama, 2630 Sugitani, Toyama 930-0194, Japan; 3 Division of Animal Experimental Laboratory, Life Science Research Center, University of Toyama, 2630 Sugitani, Toyama 930-0194, Japan; 4 Department of Neuroscience, Jikei University School of Medicine, Tokyo 105-8461, Japan

## Abstract

Behavioural tagging is the transformation of a short-term memory, induced by a weak experience, into a long-term memory (LTM) due to the temporal association with a novel experience. The mechanism by which neuronal ensembles, each carrying a memory engram of one of the experiences, interact to achieve behavioural tagging is unknown. Here we show that retrieval of a LTM formed by behavioural tagging of a weak experience depends on the degree of overlap with the neuronal ensemble corresponding to a novel experience. The numbers of neurons activated by weak training in a novel object recognition (NOR) task and by a novel context exploration (NCE) task, denoted as overlapping neurons, increases in the hippocampal CA1 when behavioural tagging is successfully achieved. Optical silencing of an NCE-related ensemble suppresses NOR–LTM retrieval. Thus, a population of cells recruited by NOR is tagged and then preferentially incorporated into the memory trace for NCE to achieve behavioural tagging.

## Abstract (web summary)

Short-term memories (STM) can become long-term memories when occurring alongside novel experiences. Here, the authors investigate the neural mechanisms behind such 'behavioural tagging' and find STM neural populations are preferentially incorporated into the ensembles encoding novel experiences.

## Graphical abstract

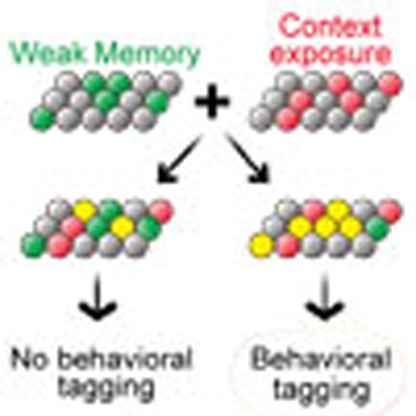

Petty daily experiences are sometimes consolidated into long-lasting memories when surprising events happen within a short time interval^1^,^2^. This phenomenon, known as behavioural tagging, can be studied in an animal model. When a weak form of behavioural training, which usually elicits only short-term memories (STMs), is temporally associated with a novel event, such as exposure to a novel environment, the weak training results in the formation of a long-term memory (LTM)^3^,^4^,^5^.

The neuronal mechanism thought to underlie behavioural tagging is called synaptic tagging and capture^3^,^4^,^5^,^6^. This mechanism was hypothesized to explain the synapse-specific nature of the late phase of long-term potentiation (L-LTP)^7^,^8^,^9^,^10^, which requires *de novo* protein synthesis^11^,^12^. This hypothesis demonstrates that during L-LTP, newly synthesized, plasticity-related proteins, which are required for stabilizing synaptic changes, are available only to the tagged synapses after unspecific transport along the dendrites from the soma^13^,^14^. Thus, the early LTP that is induced, for example, by a weak tetanic stimulation persists over time when a separate pathway receives a strong tetanic stimulation in two pathway experiments; L-LTP is induced because plasticity-related proteins are captured by the synapses tagged by the weak tetanic stimulation^13^,^14^,^15^.

Recent studies revealed that memory is represented in the brain via the activity of a neural ensemble activated during learning^6^,^16^,^17^,^18^,^19^. Individual memories are encoded by different neuronal ensembles. Overlap in the population of neurons activated by a conditioned stimulus and an unconditioned one was observed in Pavlovian conditioning, such as fear conditioning and conditioned taste aversion training^20^,^21^. According to the synaptic tagging and capture hypothesis, two events (for example, one weak and one strong input) converge in a common population of neurons where the synaptic tag and the plasticity-related proteins interact^7^,^8^,^9^,^10^. However, whether a shared population of neurons for STM and LTM is critical for behavioural tagging is unknown. Here we show that the recall of the LTM of a weak training, produced by behavioural tagging, depends on the neuronal ensemble recruited by the novel event. These results suggest that sharing a neuronal ensemble is important for behavioural tagging.

## Results

### NOR–STM is transformed into a LTM through behavioural tagging

**Figure 1:**
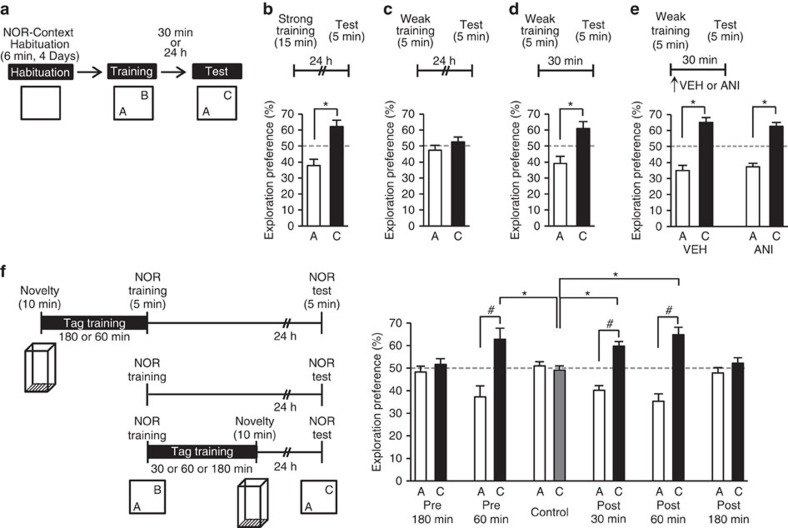
Novel context exploration (NCE) training in a narrow time window leads to NOR–LTM formation. (**a**) Novel object recognition (NOR) set-up. Mice were habituated to the NOR arena in the absence of objects for 6 min per day for 4 consecutive days, after which they were exposed for 5 min (weak training) or 15 min (strong training) in the same arena to two objects (A and B). Then, after 30 min or 24 h, they underwent a 5 min retention test in which one of the objects (B) was replaced with a novel object (C). (**b**–**e**) Animals' exploration preference for the familiar or novel object in a memory retention test (percent of time exploring each object). Bars above each graph indicate the time interval between training and test. An asterisk indicates a significant difference between the two preferences. Data are presented as mean±s.e.m. VEH, vehicle; ANI, anisomycin. (**b**) NOR–LTM was formed by training for 15 min (*n*=5; *t*-test, *t*_8_=−4.402, *P*=0.002). (**c**) NOR–LTM was not formed by training for 5 min (*n*=5; *t*-test, *t*_8_=−1.203, *P*=0.263). (**d**) NOR–STM was formed by training for 5 min (*n*=9; *t*-test, *t*_16_=−3.694, *P*=0.001). (**e**) NOR–STM formed by training for 5 min was not affected by protein synthesis inhibitor (VEH: *n*=10; *t*-test, *t*_18_=−6.799, *P*=2.3E-06; ANI: *n*=7; *t*-test, *t*_12_=−8.034, *P*=3.6E-06; VEH versus ANI for exploration preference for C in test: *t*-test, *t*_15_=0.551, *P*=0.589). There was no significant difference between any of the groups in total exploration time at training (*t*-test, *t*_15_=−0.071, *P*<0.943). (**f**) Effect of NCE on the retention of NOR–LTM. Left, behavioural tag-training scheme. Right, exploration preference for familiar and novel objects in test sessions (Pre 180 min: *n*=8; *t*-test, *t*_14_=−0.953, *P*=0.350; Pre 60 min: *n*=5; *t*-test, *t*_8_=−3.683, *P*=0.006; Control: *n*=25; *t*-test, *t*_48_=0.304, *P*=0.762; Post 30 min: *n*=8: *t*-test, *t*_14_=−6.880, *P*=7.5E-06; Post 60 min: *n*=9: *t* test, t_16_=−6.160, *P*=1.3E-05; Post 180 min: *n*=14; *t*-test, *t*_26_=−1.290, *P*=0.208). Control, no NCE training control. There was no significant difference between any of the groups for total exploration time at training (one-way ANOVA: *F*_5,68_=0.673, *P*=0.645). The number sign (#) and asterisk indicate a significant difference (*P<0.05, ^#^*P*<0.05). Data are presented as mean±s.e.m. Error bars indicate s.e.m. *n*, number of animals.

We first established a behavioural tagging paradigm in mice using two tasks: NOR and novel context exploration (NCE) (Fig. 1). Strong training (exploring two objects for 15 min) generated a LTM that lasted for at least 24 h in NOR (Fig. 1b). Weak training (exploring for 5 min) generated a STM that lasted for 30 min, but was lost by 24 h (Fig. 1c,d). The STM was independent of protein synthesis, as infusion of anisomycin, a protein synthesis inhibitor, into the hippocampal CA1 region did not affect memory retention (Fig. 1e and Supplementary Fig. 1). NCE training (exposure to a novel context for 10 min) produced a LTM for context^22^ (Supplementary Fig. 2A). Importantly, when NCE training was done either 60 min before or 30 or 60 min after NOR, the NOR-induced STM was transformed into a LTM (one-way analysis of variance (ANOVA) of the preference for object C during the retention test: *F*_5,68_=6.337, *P*=7.9E-10; Tukey–Kramer *post hoc* test for control versus 60 min before, or 30 or 60 min after NOR, *P<0.05; unpaired *t*-test for the preference for A versus C: Control, *t*_30_=0.621, *P*=0.539; 60 min before NOR, *t*_8_=−3.683, *P*=0.006; 30 min after NOR, *t*_14_=-6.880, *P*=7.5E-06; 60 min after NOR, *t*_16_=-6.160, *P*=1.3E-05; Fig. 1f). This transformation did not occur if the NCE training was done 180 min before or 180 min after NOR. The LTM for the novel context was not affected by the weak NOR training (Supplementary Fig. 2B,C).

**Figure 2:**
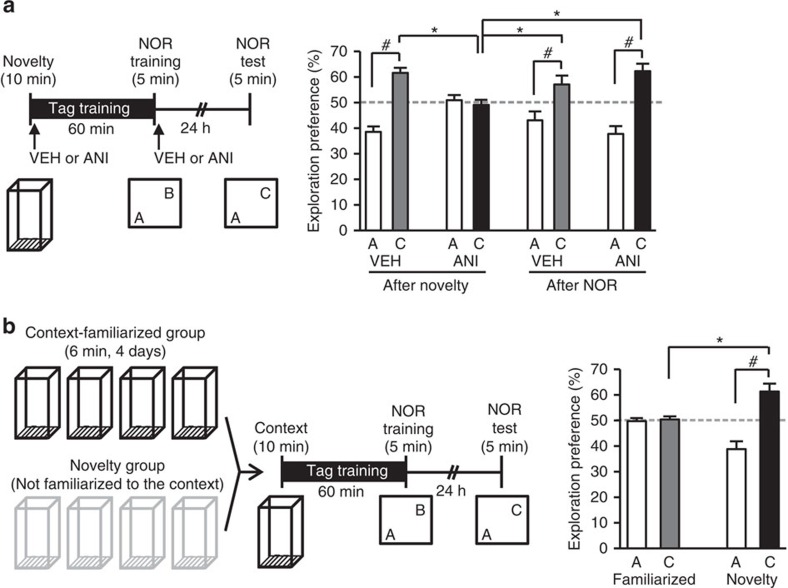
Place novelty promotes NOR–LTM dependently of hippocampal protein synthesis. (**a**–**b**) Exploration preferences for familiar (A) and novel (C) objects in test session. Behavioural tag-training involved NCE (10 min) and then, after 60 min, NOR training (5 min). (**a**) Left, behavioural paradigm. ANI was injected into the hippocampus immediately after the NCE or NOR training. Right, effect of ANI on NOR–LTM (VEH after novelty: *n*=9; *t*-test, *t*_16_=−8.637, *P*=2E-07. ANI after novelty: *n*=9; *t*-test, *t*_16_=0.661, *P*=0.517. VEH after NOR: *n*=10; *t*-test, *t*_18_=−4.150, *P*=0.0006; ANI after NOR, *n*=9, *t*-test *t*_16_=−5.837, *P*=2.5E-05; two-way ANOVA of the preference for object C during the retention test: drug, *F*_1,33_=3.518, *P*=0.069; time, *F*_1,33_=3.716, *P*=0.062; Drug versus time interaction, *F*_1,33_=10.39, *P*=0.002 (Tukey–Kramer *post hoc* test), * *P*<0.05). There was no significant difference between any of the groups for total exploration time at training (two-way ANOVA of total exploration time at training: drug, *F*_1,33_=0.411, *P*=0.525; time, *F*_1,33_=0.388, *P*=0.537; Drug versus time interaction, *F*_1,33_=1.687, *P*=0.203). (**b**) Left, behavioural paradigm. Mice were familiarized with the square chamber for 6 min per day for 4 consecutive days before the onset of behavioural tag-training. Right, effect of the familiarization on NOR–LTM (familiarized: *n*=10; *t*-test, *t*_18_=−0.330, *P*=0.744; novelty: *n*=9; *t*-test, *t*_16_=−5.219, *P*=8.4E-05; *t*-test for exploration preference for C between familiarized and novelty, *t*_17_=3.462, *P*=0.002). There was no significant difference between any of the groups for total exploration time at training (*t*-test *t*_17_=0.929, *P*=0.380). Data are presented as mean±s.e.m. of the percentage of time exploring a particular object out of the total time of object exploration. ^#^ and * indicate a significant difference (*P<0.05, ^#^*P*<0.05). Error bars indicate s.e.m. *n*, number of animals.

Anisomycin application to CA1 inhibited this transformation when injected immediately after NCE, but not after NOR training (two-way ANOVA for the preference for object C during the retention test: drug versus time interaction, *F*_1,33_=10.39, *P*=0.002; Fig. 2a and Supplementary Fig. 3). Exposure to a familiar context failed to transform the NOR-induced STM into a LTM even when done 60 min before NOR training (Fig. 2b). Thus, proteins synthesized in response to NCE prolonged the storage of NOR memory acquired during a critical time window when NCE preceded NOR training.

### Overlapping neurons increase during behavioural tag-training

**Figure 3:**
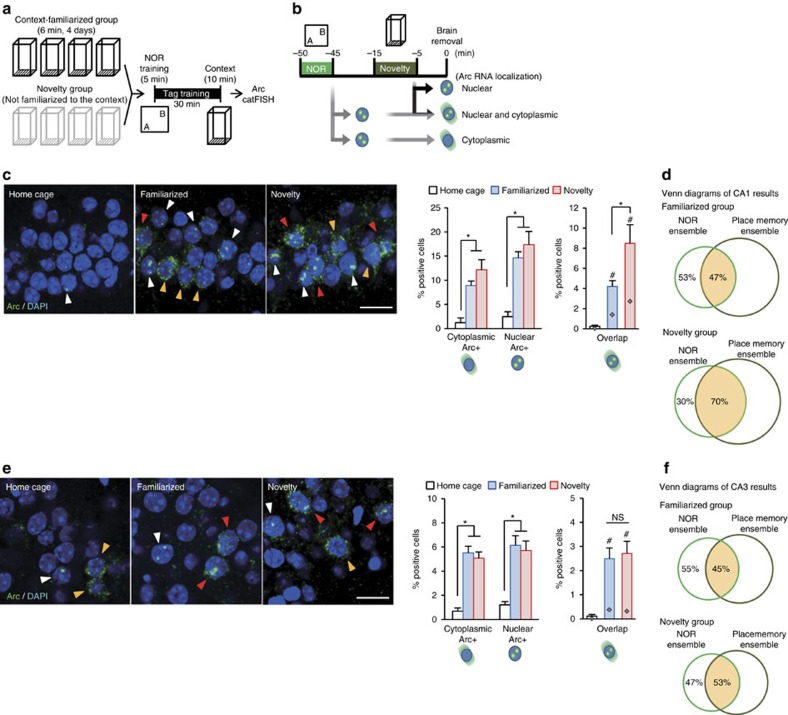
Cell ensemble analyses after behavioural tag-training in hippocampal subregions. (**a**) catFISH experiment scheme. Control mice were familiarized to the square chamber and subjected to behavioural tag-training. Mice were killed 5 min after the behavioural session. Although non-behavioural tag group done outside the temporal proximity critical to tagging (that is, 180 min) may also serve as a proper control, the cytoplasmic Arc RNA signal disappears 60 min after transcription^23^, which made it impossible to carry out the catFISH experiment outside the temporal proximity critical to tagging. (**b**) Scheme of intracellular localization of *Arc* RNA. (**c**–**f**) Detection of cells activated during behavioural tag-training. Left panels, representative photomicrographs of *Arc* signals in slices from CA1 (**c**) and CA3 (**e**) of home cage, familiarized and novelty groups. The *Arc* RNA signal and DAPI nuclear staining are shown in green and blue, respectively. Scale bar, 25 μm. Middle graphs show the percentages of cells containing cytoplasmic (yellow arrowheads) or nuclear (white arrowheads) *Arc* RNA in DAPI-positive cells in CA1 (**c**) and CA3 (**e**). Right panels show the percentages of cytoplasmic and nuclear *Arc* RNA double-positive cells (red arrowheads) in CA1 (**c**) and CA3 (**e**). Chance levels for double-positive cells are also shown in the graph (grey diamonds). Familiarized and novelty groups showed higher percentages of cytoplasmic or nuclear *Arc* RNA-positive cells in CA1 and CA3 than did the home cage group, but both percentages were comparable between familiarized and novelty groups (home cage, *n*=9; familiarized, *n*=11; novelty, *n*=12; one-way ANOVA, % CA1 cytoplasmic: *F*_2,31_=13.04, *P*=9.1E-05; % CA1 nuclear: *F*_2,31_=15.38, *P*=2.8E-05; % CA3 cytoplasmic: *F*_2,31_=27.55, *P*=2E-07; % CA3 nuclear: *F*_2,31_=13.45, *P*=7.3E-05, with Tukey–Kramer *post hoc* tests, * *P*<0.05). ^#^ indicates a significant difference between percentage of overlap and its chance level in each group (CA1: home cage, *t*-test *t*_16_=1.954, *P*=0.068; familiarized, *t*-test *t*_20_=4.567, *P*=0.0001; novelty, *t*-test *t*_22_=2.781, *P*=0.010; CA3: home cage, *t*-test *t*_16_=1.720, *P*=0.104; familiarized, *t*-test *t*_20_=4.699, *P*=0.0001; novelty, *t*-test *t*_22_=4.734, *P*=0.0001, ^#^*P*<0.05). The size of each circle reflects the cell number. *n*, number of animals; three sections were analysed from each animal. Data are presented as mean±s.e.m. Error bars indicate s.e.m. NS, not significant. Venn diagram showing percent overlap between NOR and NCE (place memory ensemble) in CA1 (**d**) and CA3 (**f**).

To identify ensembles that are activated during behavioural tag-training, we performed cell compartment analysis of temporal activity using fluorescence *in situ* hybridization (catFISH)^23^ (Fig. 3). This assay detects the temporal activation of neurons, based on the intracellular localization of RNA for *Arc*, a gene expressed early after activation: *Arc* RNA is found in the nucleus 5 min after its transcriptional induction, while it localizes in the cytoplasm 25–35 min after induction^23^. The detection of cytoplasmic and nuclear *Arc* RNA distinguished neuronal populations engaged by NOR and NCE, respectively (Fig. 3a,b). The proportions of cytoplasmic and nuclear *Arc*-positive cells in the hippocampal CA1 region were similar after novelty tag-training and familiar tag-training (Fig. 3c). More cells were positive for nuclear *Arc* than for cytoplasmic *Arc*. The most prominent result was a higher percentage of double-positive cells (that is, *Arc* signals detected in both the cytoplasm and the nucleus, denoted here as ‘overlapping neurons') after novelty tag-training than familiar tag-training (ANOVA of CA1 double-positive cells: *F*_2,31_=10.39, *P*=0.0003; Tukey–Kramer *post hoc* test for novelty-tag versus familiar-tag, *P*<0.05). Seventy percent of the NOR ensemble was shared by the context (place memory) ensemble in the novelty group, while 47% of the NOR ensemble was overlapped by the context (place memory) ensemble in the familiarized group (Fig. 3d). These results suggest that sharing an activated population of neurons underlies the successful behavioural tagging. In the CA3 region, the percentages of overlapping neurons between the novelty and the familiarized groups were comparable (ANOVA of CA3 double-positive cells: *F*_2,31_=10.61, *P*=0.003; Tukey–Kramer *post hoc* test for novelty-tag versus familiar-tag, *P*=0.922; Fig. 3e,f). Similar results were obtained in the dentate gyrus (DG) region (ANOVA of DG double-positive cells: *F*_2,22_=5.950, *P*=0.009; Tukey–Kramer *post hoc* test for novelty-tag versus familiar-tag, *P*=0.651; Supplementary Fig. 4A,B). These findings suggest that inputs from two different sources of information, NOR and NCE, converged at the same neuronal ensemble in CA1 when behavioural tagging was successfully achieved.

### Retrieval of NOR–LTM depends on the initial context-ensemble

**Figure 4:**
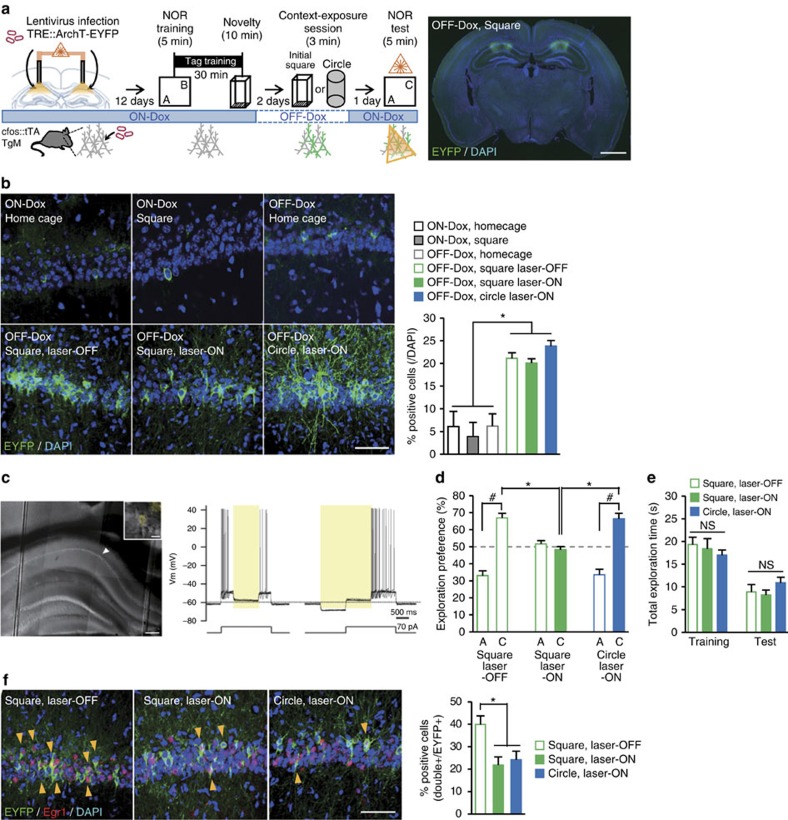
Optogenetic silencing of the cell ensembles corresponding to the initial place impairs NOR memory retrieval. (**a**) Left, optogenetic experimental scheme. The activity-dependent targeting of neurons in c-fos-tetracycline transactivator (c-fos::tTA) mice treated with a lentivirus (LV) harbouring a TRE upstream of an archaerhodopsin-T and enhanced yellow fluorescent protein (ArchT–EYFP) fusion gene. The horizontal blue line indicates the presence of Dox. Twelve days after LV infection, c-fos::tTA mice underwent behavioural tag-training consisting of NOR, followed by NCE. After 2 days of OFF-Dox, mice were subjected to context-exposure session consisting of exposure to a square or circular chamber under OFF-Dox conditions. ArchT–EYFP expression was induced in activated cells (green). On the next day, mice were subjected to the NOR test session under ON-Dox conditions, with or without laser illumination in the hippocampus, and then were killed for immunohistochemistry. Right, representative ArchT–EYFP labelling pattern of CA1 cells in a LV-injected c-fos::tTA mouse that had been exposed to the square chamber OFF-Dox. The EYFP (green) and DAPI (blue) signals were visualized by immunostaining for EYFP followed by fluorescence microscopy. Scale bar, 1 mm. (**b**) Activity-dependent and OFF-Dox-dependent labelling of cells with ArchT–EYFP in the CA1 region. Left, representative photomicrographs showing ArchT–EYFP labelling patterns in CA1 of LV-injected c-fos::tTA mice obtained in different conditions of context-exposure session (square, circular or home cage) and illumination (Laser ON and Laser OFF). Square, square chamber; circle, circular chamber. Scale bar, 50 μm. Right, percentages of ArchT–EYFP-positive cells in the CA1 region normalized to DAPI-positive cells. Exposure to the chambers without Dox treatment induced the larger ArchT–EYFP expression compared with either the home cage or ON-Dox conditions (ON-Dox home cage, *n*=5; ON-Dox square, *n*=3; OFF-Dox home cage, *n*=4; OFF-Dox square laser OFF, *n*=9; OFF-Dox square laser ON, *n*=11; OFF-Dox circle laser ON, *n*=11; two-way ANOVA for the percentages of ArchT–EYFP-positive cells: Dox treatment versus context-exposure interaction, *F*_1,39_=18.44, *P*=0.0001; Dox treatment, *F*_1,39_=18.91, *P*=0.0001; Context-exposure, *F*_1,39_=10.33, *P*=0.002 (with the Tukey–Kramer *post hoc* test); * *P*<0.05). *n*, number of animals; three sections were analysed from each animal. Data are presented as mean±s.e.m. Error bars indicate s.e.m. (**c**) Hyperpolarization and suppression of spiking by light-emitting diode illumination in a CA1 neuron of LV-injected c-fos::tTA mice. Left, low and high magnification images of the slice and the neuron recorded (inset). The arrowhead indicates the tip of the recording pipette. Scale bar, 200 μm, 10 μm (inset). Right, the membrane potentials of the neuron (above) and the injected current (below). The yellow boxes indicate the periods of yellow light-emitting diode illumination. Horizontal dotted line shows −60 mV. (**d**) Silencing of initial chamber-related cell ensembles impairs NOR–LTM retrieval (square laser OFF, *n*=9, *t*-test *t*_16_=−8.780, *P*=1.6E-07; square laser ON, *n*=11, *t*-test *t*_20_=1.364, *P*=0.187; circle laser ON, *n*=11, *t*-test *t*_20_=−7.330, *P*=4.4E-07; one-way ANOVA, *F*_2,30_=16.85, *P*=2E-05, with Tukey–Kramer *post hoc* tests, *P<0.05). (**e**) Optogenetic manipulation did not affect the exploration behaviour (one-way ANOVA, in training: *F*_2,30_=0.444, *P*=0.646; in test: *F*_2,30_=1.167, *P*=0.326). (**f**) Laser illumination inhibited *Egr1* expression in ArchT–EYFP-positive cells 60 min after NOR memory retrieval. Left, representative photomicrographs of triple immunofluorescence for EYFP (green), Egr1 (red) and DAPI (blue). Yellow arrowheads indicate Egr1 and EYFP double-positive cells. Scale bar, 50 μm. Right, percentages of Egr1 and EYFP double-positive cells normalized to EYFP-positive cells in the CA1 region (one-way ANOVA, *F*_2,30_=6.439, *P*=0.005, with Tukey–Kramer *post hoc* test, *P<0.05). ^#^ and * indicate significant differences (* *P*<0.05, ^#^ *P*<0.05). Error bars indicate s.e.m. NS, not significant.

To molecularly detect and manipulate the population of neurons activated during the retrieval of memories of specific events, we employed c-fos::tetracycline transactivator (c-fos::tTA) transgenic mice. In a series of optogenetic experiments, we injected, into the CA1 region, a recombinant lentivirus (LV) harbouring a tetracycline response element (TRE) upstream of a fusion gene expressing enhanced archaerhodopsin-T 3.0 (ArchT) and enhanced yellow fluorescent protein (EYFP) (Fig. 4a and Supplementary Fig. 5). In these transgenic mice, neuronal activity drives the promoter of the immediate-early gene c-fos, and tTA is expressed; tTA binds to the TRE and, in the absence of doxycycline (Dox), drives the expression of archaerhodopsin-T and enhanced yellow fluorescent protein (ArchT–EYFP). ArchT is a light-activated proton pomp that hyperpolarizes cells and silences their neuronal firing^24^. Therefore, upon memory recall, the population of activated neurons expressed ArchT–EYFP protein 2 days after the withdrawal of Dox (OFF-Dox) in a context-exposure-dependent manner (two-way ANOVA: Dox treatment versus context-exposure interaction, *F*_1,39_=18.44, *P*=0.0001; Fig. 4b). Recording of membrane potentials from neurons in an acute hippocampal slice revealed a robust firing, elicited by a current injection, which was completely blocked by delivery of yellow light (565 nm) that activated ArchT of CA1 cell expressing ArchT–EYFP (Fig. 4c).

We then examined whether the silencing of ensembles corresponding to the NCE suppresses the retrieval of a NOR memory. c-fos::tTA mice were treated with the LV bilaterally in CA1 and allowed 12 days to recover in the presence of Dox (ON-Dox) (Fig. 4a). The mice were then subjected to the behavioural tag-training and taken off Dox to allow for neuronal activity-dependent transgene expression. After 2 days, mice were exposed to a familiar square context or to a novel circular context to induce ArchT–EYFP expression in activated cells (context-exposure session). The next day, with ON-Dox, optical fibres were inserted into CA1 and mice were tested for NOR memory during which laser light (589 nm) was delivered to CA1. Mice re-exposed to the square context showed no preference for a novel object over a familiar object (square laser ON) (unpaired *t*-test for the preference for A versus C: square laser ON, *t*_20_=1.364, *P*=0.187; Fig. 4d). Control mice, which were fitted with optical fibres but did not receive the laser light, showed normal NOR memory as they had a higher preference for the novel object (square laser OFF). Similarly, mice exposed to the circular context spent significantly more time exploring the novel object (circle laser ON) (unpaired *t*-test for the preference for A versus C: square laser OFF, *t*_16_=−8.780, *P*=1.6E-07; circle laser ON, *t*_20_=−7.325, *P*=4.4E-07; ANOVA of preference for C in test: *F*_2,30_=16.85, *P*=2E-05; Tukey–Kramer *post hoc* test square laser OFF and circle laser ON versus square laser ON, *P*<0.05; Fig. 4d). There was no significant difference between any of the groups for total exploration time in the training or test sessions (Fig. 4e). Light stimulation suppressed the cellular activity of ArchT-positive cells that had been induced by context-exposure, as the percentage of EYFP and *Egr1* (an immediate-early gene) double-positive cells was significantly less in the laser ON group (Fig. 4f). Pearson correlation analysis in square-labelled group containing both laser-OFF and -ON groups showed the significant correlation between the overlapping and memory performance (*r*=0.426, *P*=0.001) (Supplementary Fig. 6). Thus, silencing of the cellular ensemble that had been activated during the retrieval of the initial context memory suppressed the retrieval of NOR memory.

### Overlap in ensembles during retrieval

**Figure 5:**
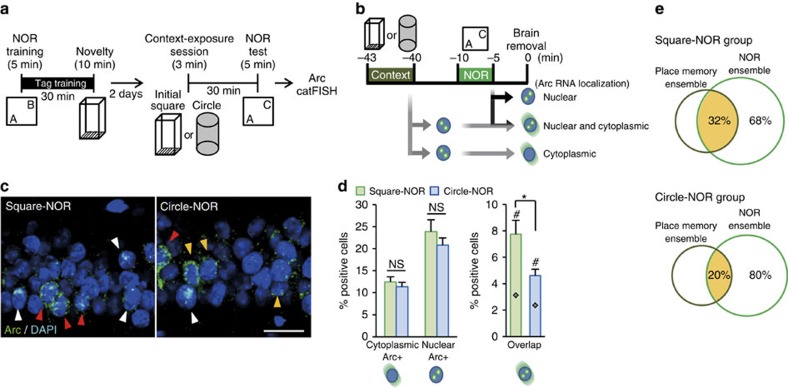
Cell ensemble analyses in CA1 after the retrieval test. (**a**) catFISH experiment scheme. Two days after behavioural tag-training, mice were exposed to a square or circular chamber followed by NOR testing at an interval of 30 min (Square-NOR or Circle-NOR, respectively), and then killed 5 min after the behavioural session. (**b**) Scheme of intracellular *Arc* RNA localization after the retrieval test. (**c**,**d**) Detection of cells activated during the behavioural session. (**c**) Representative photomicrographs of *Arc* signals in slices from the CA1 of Square-NOR or Circle-NOR groups. The *Arc* RNA signal and DAPI nuclear staining are shown in green and blue, respectively. Scale bar, 25 μm. (**d**) Left graph shows the percentage of cells containing cytoplasmic (yellow arrowheads in **c**) or nuclear (white arrowheads in **c**) *Arc* RNA in DAPI-positive cells. Right graph shows the percentage of cytoplasmic and nuclear *Arc* RNA-double-positive cells (red arrowheads in **c**). Chance level for double-positive cells (grey diamonds) is also shown. The percentages of cells positive for cytoplasmic or nuclear *Arc* RNA were comparable between Square-NOR and Circle-NOR groups (Square-NOR, *n*=9; Circle-NOR, *n*=9; % cytoplasmic, *t*-test *t*_16_=0.709, *P*=0.488; % nuclear, *t*-test *t*_16_=0.981, *P*=0.341). ^#^ indicates significant difference between the percentage of overlap and the chance level in each group (Square-NOR, *t*-test *t*_16_=3.820, *P*=0.001; Circle-NOR, *t*-test *t*_16_=4.157, *P*=0.0007). * indicates a significant difference (*P*<0.05) with unpaired Student's *t*-test. *n*, number of animals; three sections were analysed from each animal. Data are presented as mean±s.e.m. Error bars indicate s.e.m. NS, not significant. (**e**) Venn diagram showing percent overlap between NOR and place memory ensemble in CA1. Size of each circle reflects the cell number.

The results of the optogenetics experiments suggest that the sharing of the CA1 ensembles observed during behavioural tag-training (see Fig. 3c) was maintained at the retrieval test. Animals were trained with behavioural tagging (NOR–STM training and NCE) and 2 days later were exposed to the initial square context or to a novel circular context, followed by NOR testing (Fig. 5a,b). catFISH revealed that the numbers of cells positive for cytoplasmic or nuclear *Arc* RNA in CA1 were comparable between the two groups of animals (Fig. 5c,d). On the other hand, the square context group had significantly more double-positive cells than did the circular context group, indicating that the CA1 ensemble sharing memory traces was maintained (unpaired *t*-test for CA1 double-positive cells, initial square context versus circular context: *t*_16_=2.718, *P*=0.015; Fig. 5c,d,e). Taken together, these results suggest that the sharing of cellular ensembles between NOR and NCE underlies the mechanism of behavioural tagging.

### Dopaminergic modulation

**Figure 6:**
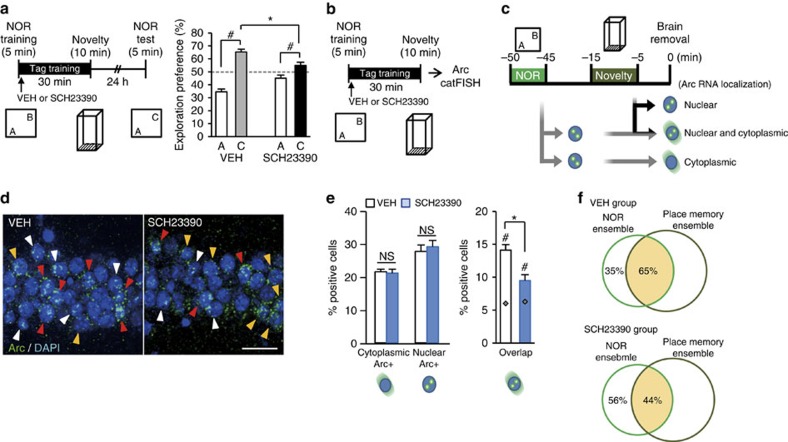
Blockade of dopamine D1/D5 receptor during behavioural tag-training affects the NOR–LTM and the ratio of overlapping cells in CA1. (**a**) Left, behavioural experimental scheme. Dopamine D1/D5 receptor antagonist SCH23390 was intraperitoneally injected into the mice immediately after the NOR training, followed by NCE at an interval of 30 min. Right, effect of the SCH23390 on NOR–LTM. Exploration preferences for familiar (A) and novel (C) objects in a test session (VEH, *n*=7, *t*-test *t*_12_=−10.78, *P*=1.5E-07; SCH23390, *n*=9, *t*-test *t*_16_=−2.816, *P*=0.012). There was no significant difference between any of the groups for total exploration time at training (*t*-test *t*_14_=−0.135, *P*=0.894). Data are presented as mean±s.e.m. of the percentage of time exploring a particular object over the total time of object exploration. ^#^, significant difference between familiar and novel objects in each group. *, significant difference between groups in exploration preference for C in test session. Error bars indicate s.e.m. (**b**) catFISH experiment scheme. Mice were subjected to behavioural tag-training as shown in **a** and then killed 5 min after the behavioural session. (**c**) Scheme of intracellular *Arc* RNA localization after behavioural tag-training. (**d**) Representative photomicrographs of *Arc* signals in slices from the CA1 of VEH or SCH23390 group. The *Arc* RNA signal and DAPI nuclear staining are shown in green and blue, respectively. Scale bar, 25 μm. (**e**) Left graph shows the percentages of cells containing cytoplasmic (yellow arrowheads in **d**) or nuclear (white arrowheads in **d**) *Arc* RNA in DAPI-positive cells. Right graph shows the percentage of cytoplasmic and nuclear *Arc* RNA double-positive cells (red arrowheads in **d**) in the CA1 region. Chance level for double-positive cells (grey diamonds) is also shown. The percentages of cells positive for cytoplasmic or nuclear *Arc* RNA were comparable between the VEH and SCH23390 groups (VEH, *n*=7; SCH23390, *n*=7; % cytoplasmic, *t*-test *t*_12_=0.318, *P*=0.755; % nuclear, *t*-test *t*_12_=−0.522, *P*=0.610). ^#^ indicates a significant difference (*P*<0.05) between the percentage overlap and the chance level in each group (VEH, *t*-test *t*_12_=8.117, *P*=3.2E-06; SCH23390, *t*-test *t*_12_=2.852, *P*=0.014). *indicates a significant difference (*P*<0.05) with unpaired Student's *t*-test. *n*, number of animals; three sections were analysed from each animal. Data are presented as mean±s.e.m. Error bars indicate s.e.m. NS, not significant. (**f**) Venn diagram showing percent overlap between NOR and NCE (place memory ensemble) in CA1. Size of each circle reflects the cell number.

Dopamine, via the D1/D5 receptor, modulates synaptic tagging and capture in two pathway experiments^25^,^26^,^27^ and also in behavioural tagging^3^,^4^,^5^. Administration of SCH23390, a D1/D5 receptor antagonist, immediately after NOR training suppressed behavioural tagging but not NOR–STM retention (Supplementary Fig. 7), as the animals' exploration preference for the novel object was significantly less than that of the control group (unpaired *t*-test, vehicle (VEH) versus SCH23390 for the exploration preference for C, *t*_14_=3.120, *P*=0.007; Fig. 6a). catFISH analysis revealed significantly fewer overlapping neurons in the SCH23390-treated group than in the saline-treated control group (unpaired *t*-test for CA1 double-positive cells initial VEH versus SCH23390: *t*_12_=3.732, *P*=0.002; Fig. 6b–f).

## Discussion

In this study, we established a behavioural tagging paradigm in mice that has several characteristics similar to one previously described in rats^3^,^4^,^5^. In our experiments, the population of overlapping neurons increased in CA1 during behavioural tag-training and also during the memory retrieval session. These results fit with the previous report showing that activity of CA1, but not CA3, increases in the presence of generalized novelty in the object-place recognition task, which suggests that novelty responses emerge within CA1, or through projection from entorhinal cortex to CA1 (ref. 28). This increase in overlap implies that a population of neurons activated during NOR training is temporally tagged (cellular tagging) and then preferentially incorporated into the cellular ensemble of NCE when the later event happens within a time frame of 60 min. The memory trace is preferentially allocated to a subset of neurons with high excitability at the time of training^29^,^30^. Neuronal excitability is temporally enhanced by learning^31^,^32^,^33^. A subpopulation of CA1 neurons activated during NOR training could be more excitable and easily re-activated during NCE, and may serve as a cellular tag, thus leading to the increase in the population of overlapping neurons. A similar mechanism could operate when NCE precedes NOR training, where a subset of NCE-activated cells is tagged and preferentially incorporated into the NOR ensemble. On the other hand, the neuronal ensemble for context has been tightly pre-fixed in the familiarized group because of the repeated pre-exposure to the context. This may prevent ensemble sharing with NOR even if neurons of the NOR ensemble have high excitability.

Inhibition of the dopamine D1/D5 receptor suppressed the behavioural tagging and the increase in the population of overlapping neurons in CA1 (Fig. 6). Previous report suggests that hippocampus and the midbrain dopaminergic neurons of the ventral tegmental area (VTA) form a functional loop, where hippocampus propagates novelty signal to the VTA when the hippocampus detects novel information^34^. Novelty exposure increases the firing rate of VTA dopaminergic neurons, which seems to be informed by hippocampal input^35^. Exposure to a novel context, but not to a familiar context, increases dopamine concentration temporally in the hippocampus in mice^36^,^37^. Moreover, dopamine acting via the D1/D5 receptor modulates the intrinsic excitability of CA1 neurons^38^. Thus, suppression of cell excitability by inhibition of dopamine, which is released from VTA neurons upon novelty exposure, may explain the observed decrease in the population of overlapping neurons and impairment of behavioural tagging.

A similar increase in the overlap between cellular ensembles was found in the association of conditioned and unconditioned stimuli in fear conditioning and conditioned taste aversion^20^,^21^. Overlap in cellular ensembles was also observed in hippocampal slices^39^. Distinct CA1 ensembles recruited by distinct afferent fibres can merge to increase the overlapping ratio of these ensembles when simultaneous activation of these distinct afferent fibres induces associative LTP. Furthermore, a recent calcium imaging study quantified similar overlap in CA1 *in vivo*, and showed that the population of overlapping in cellular ensemble between distinct events within the same day has ability to associate these events^40^. Sharing the cellular ensemble may be a general mechanism underlying the interaction of distinct memories.

Similarities between synaptic tagging and capture and behavioural tagging, such as dependence on protein synthesis and dopamine and the characteristic time window^3^,^4^,^5^,^7^,^25^, suggest that the former mechanism underlies behavioural tagging. In the overlapping neurons, plasticity-related proteins induced by NCE could be recruited to synapses involved in the cellular ensemble that constitutes the NOR memory trace, which then helps the NOR-induced STM to be consolidated into LTM. In this way, the overlapping neurons may be important for behavioural tagging to be established. This idea is supported by our optogenetic experiments showing that silencing of the neuronal ensemble corresponding to the initial context impaired the retrieval of NOR–LTM. The different sizes of the neuronal populations that were optically silenced (32% and 20% of NOR memory trace for square-NOR and circular-NOR, respectively, Fig. 5e) do not simply account for the different effects on behavioural tagging because silencing 20% of the NOR memory trace had no effect at all on the behavioural tagging in the circular-NOR group (Fig. 4d). There may be a non-linear relationship between the degree of overlap and the establishment of behavioural tagging, in which overlapped ensembles above the threshold are required for behavioural tagging to occur. Taken together, the linking of cellular ensembles, specifically the convergent activation of neuronal subpopulation for NOR and NCE, contributes to behavioural tagging.

## Methods

### Animals

All procedures involving the use of animals complied with the guidelines of the National Institutes of Health and were approved by the Animal Care and Use Committee of the University of Toyama and the Institutional Committee for the Care and Use of Experimental Animals of Jikei University. The C57BL/6J and ICR mice were purchased from Japan SLC. The c-fos::tTA mice were purchased from the Mutant Mouse Regional Resource Center (stock number: 031756-MU). Surgery was conducted on 8–16 weeks old male wild-type or c-fos::tTA mice with a C57BL/6J background.

The progeny for the c-fos-tTA line were generated using *in vitro* fertilization with eggs from C57BL/6J mice and embryo transfer techniques to produce a number of animals for behavioural analysis^21^. Genotyping was performed with PCR using genomic DNA isolated from the tails of the pups as described previously^41^.

The mice were maintained on a 12 h light–dark cycle (lights on 8:00 am) at 24±3 °C and 55±5% humidity, given food and water *ad libitum*, and housed with littermates.

### Surgery for drug microinfusion

Surgery was performed as described previously^42^. Under Nembutal anesthesia and using standard stereotactic procedures, stainless steel guide cannulas (22 gauge, Plastics One, USA) were implanted bilaterally into the dorsal hippocampus (anteroposterior (AP) 2.0 mm, mediolateral (ML) ±1.5 mm, dorsoventral (DV) 1.7 mm from bregma). Anisomycin (Sigma A9789; 62.5 μg μl^−1^), a protein synthesis inhibitor, was dissolved with 1N HCl and adjusted to pH 7.4 with NaOH in the PBS as a VEH solution. Micro-infusions into hippocampal region (0.5 μl) were made at a rate of 0.25 μl min^−1^. The injection cannula was left in place for 2 min after the infusion.

### Behavioural analysis

Male mice, approximately 8–16 weeks old, were numbered and housed in cages of three or four and randomly allocated to each experimental group before the experiment began. All training and testing was conducted during the light cycle. The soundproof experimental room for behavioural analyses adjoined the animal housing room. Animals in their home cages were placed on a desk in the animal housing room for ∼10 min before each session. Each animal was gently caught at the base of its tail and transferred to a carrier for transportation to the experimental room. New maintenance cage with bedding was used for the transportation of mice between their home cages and experimental apparatuses for both NOR and NCE (see below for details). A mesh white-plastic bowl was used for the transportation of mice between their home cages and a circular context (see Figs 4 and 5).

In the drug micro-infusion experiments, no hippocampus-injected animal was excluded from analysis, as the tip of the cannula did not reach in the hippocampus. In the virus experiments, no hippocampus-infected animals were excluded from the analysis as virus infection did not reach the hippocampus. These criteria were pre-established. No statistical method was used to pre-determine the sample sizes, as sample sizes of this work are similar to those reported in previously published papers, such as Moncada and Viola (2007; ref. 5), Ballarini *et al*. (2009; ref. 3) and Barot *et al*. (2008; ref. 20), and so on.

### NCE

NCE was performed as described previously^22^. Training and testing sessions were conducted in the experimental room. The square-type chamber had a transparent acrylic-board front wall and white side and back walls (width × depth × height: 175 × 165 × 300 mm, respectively), and the chamber floors consisted of 26 stainless steel rods with a diameter of 2 mm that were placed 5 mm apart (generally, this square-type chamber is used as a mouse contextual fear conditioning chamber). During the training session, mice were placed in the square-type chamber for 10 min and mice were then returned to their home cages. During the testing session, mice were placed back into experienced chamber for 3 min. At the end of each session, mice were returned to their home cages and chamber was cleaned with 80% ethanol. All experiments were conducted using a video tracking system (Muromachi Kikai; Japan) to measure the motility of the animals. The motility was calculated as the cumulative area of movement (pixel size) per 0.1 s in the learning and testing sessions.

### NOR test

NOR memory test was performed as described previously^43^,^44^ with some modifications to adjust for mice. Mice prefer to explore more the novel objects compared with the familiar objects. This preference is used to design a behavioural paradigm known as an object recognition task, which has been employed to evaluate recognition memory^43^,^44^. NOR was conducted in an experimental arena that had a transparent acrylic-board front wall partially covered with a white tape and grey side and back walls (width × depth × height: 290 × 250 × 290 mm, respectively), and the arena floor consisted of grey acrylic-board. Before the commencement of the behavioural experiments, mice were individually handled and habituated to the arena by allowing them to freely explore without the objects for 6 min per day for 4 days. During the training session, mice were placed in the arena with two different objects positioned in two corners 40 mm from lateral walls, and allowed to explore the objects for 5 or 15 min. Memory test was carried out at 30 min (see Fig. 1d,e and Supplementary Fig. 6), 24 h (see Fig. 1b,c,f, Fig. 2a,b and Fig. 6a), 48 h (see Fig. 5a) or 72 h (see Fig. 4d) later. During the test session, mice explored the arena for 5 min, in which one familiar object was replaced with a novel object. The objects were made of yellow-plastic (stamp: 40 × 40 × 40 mm), black-ceramic (elephant-shaped salt case: 75 × 50 × 40 mm) or white-ceramic (small cup: 58 mm diameter × 45 mm height). The position of the two objects (familiar and novel) were counterbalanced and randomly permuted for each animal. The arena and the objects were cleaned thoroughly with 80% ethanol between trials to wipe out these olfactory cues. Exploration was defined as sniffing or touching the objects with nose. Sitting or climbing on, or turning around the objects was not considered as exploratory behaviour. A video tracking system (Muromachi Kikai; Japan) positioned over the arena was used as a video camera, and the animal behaviour was recorded for later analysis. Time spent exploring each object was manually analysed by an observer blind to the experimental conditions and expressed as a percentage of the total exploration time.

For behavioural tag-training, NOR training for 5 min was followed by NCE with square-type chamber, and vice versa.

As a control group for behavioural tagging, mice were familiarized to the square-type chamber by allowing them to freely explore without objects for 6 min per day for 4 days.

For dopamine D1/D5 receptor antagonist treatment, mice were injected intraperitoneally with saline (VEH) or SCH23390 (0.2 mg kg^−1^ of body weight; Sigma D054) immediately after the end of NOR training, and 30 min later, mice were subjected to NCE with square-type chamber (see Fig. 6a,b).

### Fluorescent *in situ* hybridization (FISH)

After the mice were killed, the brains were rapidly extracted, frozen and stored at −80 °C for later sectioning. Coronal sections (20 μm thick) were cut using a cryostat, air-dried and mounted onto slides. Sections containing the hippocampi were selected for *in situ* hybridization. FISH was essentially performed as described previously^21^,^45^.

Images were acquired using a Zeiss LSM 780 confocal microscope with a Plan-Apochromat × 40 objective lens for CA1 and CA3 regions or × 20 objective lens for DG region. The photomultiplier tube assignments, pinhole sizes and contrast values were kept constant. Images were acquired by collecting z-stacks (1 μm thick optical sections). Using the Zen software (Zeiss), each cell was characterized through several serial sections, and only cells containing whole nuclei were included in the analysis. The details of the classification analysis were described previously^21^,^23^,^45^. Small, bright, uniformly 4,6-diamidino-2-phenylindole (DAPI)-stained nuclei (from putative glial cells) were not analysed. All other whole nuclei were analysed from top to bottom along z-stacks. The cells containing two small intense intranuclear fluorescent foci were designated nuclear-positive (Nuc) neurons. Cells containing perinuclear and cytoplasmic labelling in multiple optical sections were designated cytoplasm-positive (Cyto) neurons, and the cells containing both intranuclear and cytoplasmic arc-positive signals were designated Nuc and Cyto double Arc+. Three sections corresponding to each region of interest (ROI) (between AP 1.7 and 1.9 mm from bregma were chosen from each mouse. Cells with DAPI-positive nuclei in the ROI were analysed by an observer blind to the experimental conditions.

### Viral vectors

The pLenti-TRE3G::ArchT 3.0-EYFP plasmid was constructed by replacing the human synapsin I promoter and improved version of tetracycline-controlled transactivator (tTA) sequences in the STB plasmid^46^ with the third generation tTA-responsive (TRE3G) promoter sequence derived from pTRE3G-IRES (Clontech, 631161) and the enhanced Archaerrhodopsin from *Halorubrum* strain TP009 version 3.0 (ArchT 3.0-EYFP) sequence derived from the pLenti-CaMKIIa-eArchT 3.0-EYFP plasmid (donated from Dr K. Deisseroth, Stanford University), respectively. To replace these fragments, TRE3G and eArchT 3.0-EYFP fragments were amplified by PCR using pTRE3G-IRES and pLenti-CaMKIIa-eArchT 3.0-EYFP plasmid, respectively, as templates with the following primers (for TRE3G fragment: sense, GGGACTAGTTTCGAATTCGTCTTCAAGAATTCCTC ; antisense, GGGCCCGGGACGCGTCATATGGGGCCCACCGGTGGATCCTTTACGAGGGTAGGAAGTGG ; for eArchT 3.0-EYFP fragment: sense, CGGGATCCGCCACCATGGACCCAAT ; antisense, GCGAATTCCCGGGTTACACCTCGTTCTC ). The resulting TRE3G fragments were subcloned into the *Spe*I–*Xma*I sites of pBluescript II (SK+) (Stratagene), generating pBS-TRE3G. The resulting eArchT 3.0-EYFP fragment was subcloned into the *Bam*HI–*Xba*I sites of pBluescript II (SK+), generating pBS-ArchT 3.0-EYFP plasmid. The *Bst*BI–*Xma*I fragment from pBS-TRE3G was subcloned into the *Bst*BI–*Xma*I sites of STB, generating pLenti-TRE3G. The *Bam*HI–*Xba*I fragment of pBS-ArchT 3.0-EYFP plasmid was subcloned into the *Bam*HI–*Xba*I sites of pLenti-TRE3G, generating pLenti-TRE3G::ArchT 3.0-EYFP plasmid. The pLenti-TRE3G::ArchT 3.0-EYFP was prepared with an EndoFree Plasmid Maxi kit (Qiagen).

The LV was prepared as described previously^21^ according to the protocol from the laboratory of K. Deisseroth ( http://www.stanford.edu/group/dlab/optogenetics/expression_systems.html). The viral titer was ∼5 × 10^9^ IU ml^−1^, as described previously^21^,^47^.

### Stereotactic surgery for virus infection

Stereotacic surgery and optic fibre placement were carried out as described previously^21^. The male c-fos::tTA mice were 8–16 weeks old at the time of surgery. Mice were anaesthetized with a pentobarbital solution (80 mg kg^−1^ of body weight; intraperitoneal injection), and the fully anaesthetized mice were placed in a stereotactic apparatus (David Kopf, USA). Mice were bilaterally implanted with guide cannulas into the dorsal hippocampi that were composed of a stainless steel pipe and a plastic cannula body (Plastics One, USA, C316GS-5/SPC: internal diameter, 0.290 mm). The guide cannula targeted hippocampal CA1 regions bilaterally (AP 2.0 mm, ML±1.4 mm, DV 0.5 mm from bregma). Micro-screws were screwed into the skull near bregma and lambda, and the guide cannula was fixed on the skull with dental cement. After the implantation of the guide cannula, lentiviral vector (1 μl per injection site) was injected into the dorsal hippocampal CA1 regions through the injection cannulas connected to two Hamilton microsyringes via polyethylene tubes filled with water. The injection cannulas (Plastics One, USA, C316IS-5/SPC: outer diameter, 0.250 mm) were inserted into the guide cannulas and targeted bilateral dorsal hippocampal CA1 regions (AP 2.0 mm, ML ±1.4 mm, DV 1.5 mm from bregma). The injection speed (0.1 μl min^−1^) was controlled with a microsyringe pump (CMA 400, Harvard Apparatus). The injection cannulas were left in place for 10 min and then slowly withdrawn. After injection, internal cannulas (Plastics One, USA, C316IS-5/SPC) that targeted above of the hippocampi (AP 2.0 mm, ML ±1.4 mm, DV 0.5 mm from bregma) were inserted into the implanted guide cannulas as dummy cannulas to protect them from dust.

### Optogenetic experiments

Optogenetic experiments were carried out as described previously^21^ with modifications. The c-fos::tTA male mice were maintained after weaning on food containing 40 mg kg^−1^ Dox. The c-fos::tTA mice were 8–16 weeks old at the time of surgery. Cannula-implanted and LV-injected c-fos::tTA mice were maintained on 1,000 mg kg^−1^ Dox food pellets. Five days after surgery, they were maintained on 40 mg kg^−1^ Dox food pellets until their withdrawal of Dox administration. Mice were handled and habituated to the NOR arena for 4 days as described above. Mice were trained with a behavioural tag-training by combination of 5-min NOR training and 10-min square-type chamber exposure as a novelty at interval of 30 min as described above. Mice were then withdrawn Dox and maintained on normal food pellets in their home cage. Two days after the onset of Dox withdrawal, mice were re-exposed to the square-type chamber or exposed to the circle-type chamber for 3 min to label the cells with ArchT 3.0-EYFP (context-exposure session), and then maintained on 1,000 mg kg^−1^ Dox food pellets. The circle-type chamber was a cylindrical chamber (diameter × height: 180 × 230 mm, respectively) with a white acrylic floor and black walls covered with black tape. New maintenance cage with bedding was used for the transportation of mice to the NOR arena or the square-type chamber for NCE. A mesh white-plastic bowl was used for the transportation of mice to circle-type chamber. Square- or circle-type chambers were placed in different locations in the same experimental room.

One day after the context-exposure session, mice were anaesthetized with 3% isoflurane for placement of the optical fibre units and the dummy cannulae were removed from the guide cannulae. The optical fibre unit, composed of a plastic cannula body, was a two-branch-type unit with an optic fibre diameter of 0.250 mm (COME2-DF2–250, Lucir, Japan). The optical fibre unit was inserted into the guide cannulae, and the guide cannulae and the optical fibre unit tightly connected with an adhesive cray (Kokuyo, Japan). The tip of the optical fibre was targeted slightly above the hippocampi (AP 2.0 mm, ML ±1.4 mm, DV 1.0 mm from bregma). Mice attached with an optical fibre were then returned to their new home cages and left individually at least for 2 h until the onset of NOR test session. Immediately before the onset of NOR test, mice were moved to the experimental room, and the fibre unit connected to the mouse was attached to an optical swivel (COME2-UFC, Lucir, Japan), which was itself connected to a laser (200 mW, 589 nm, COME-LB473/200, Lucir, Japan) via a main optical fibre. The delivery of light pulses was controlled by a schedule stimulator (COME2-SPG-2, Lucir, Japan) operating in time-lapse mode. Optical illumination (continuous 589 nm light, ∼5 mW output from the fibre tip) was delivered and kept during the 5 min NOR test session, and then mice were returned to their home cage individually without removing an optic fibre. Sixty minutes after the end of the NOR test session, mice were deeply anaesthetized with an overdose of pentobarbital solution, and perfused transcardially with 4% paraformaldehyde in PBS (pH 7.4), followed by an immunohistochemical analysis to confirm the LV infection and Egr1 expression. An attached optic fibre was removed from the mice after the anesthesia.

### Immunohistochemistry

Immunohistochemistry was carried out as described previously^21^. Mice were deeply anaesthetized with an overdose of pentobarbital solution and perfused transcardially with 4% paraformaldehyde in PBS (pH 7.4). The brains were removed and further post-fixed by immersion in 4% paraformaldehyde in PBS for 24 h at 4 °C. Each brain was equilibrated in 25% sucrose in PBS for 2 days and then frozen in dry-ice powder.

For EYFP and Egr1 staining, coronal sections of 50 μm thick were cut on a cryostat and stored at −20 °C in cryoprotectant solution (25% glycerol, 30% ethylene glycol, 45% PBS, Liu *et al*.^17^) until further use. Sections were transferred to 12-well cell culture plates (Corning, Corning, NY) containing Tris-buffered saline TBS-T buffer (with 0.1% Triton X-100, 0.05% Tween-20). After washing with TBS-T buffer, the floating sections were treated with blocking buffer (5% normal donkey serum (S30, Chemicon) in TBS-T) at room temperature for 1 h. Reactions with primary antibodies were performed in blocking buffer containing rat anti-GFP (1:1000, Nacalai Tesque, 04404-84, GF090R) and/or rabbit anti-Egr1 (1:1000, Santa Cruz Bio, sc-189) antibodies at 4 °C for 1–2 nights. After three times 20 min washes with TBS-T, the sections were incubated with donkey anti-rat IgG-AlexaFluor 488 (1:1000, Molecular Probes, A21208) and/or donkey anti-rabbit IgG-AlexaFluor 546 secondary antibodies (1:1000, Molecular Probes, A10040) in the blocking buffer at room temperature for 3 h. The sections were treated with DAPI (1 μg ml^−1^, Roche Diagnostics, 10236276001) and then washed with TBS three times (20 min per wash). The sections were mounted on slide glass with ProLong Gold antifade reagent (Invitrogen). Images were acquired using a Zeiss LSM 780 confocal microscope with a Plan-Apochromat × 20 objective lens. The photomultiplier tube assignments, pinhole sizes and contrast values were kept constant. To quantify the number of EYFP-positive and/or Egr1-positive and DAPI-positive cells in the CA1 region, images of the 200 μm × 200 μm ROIs were acquired by collecting z-stacks (∼5 optical sections 5 μm thick). Three sections (between AP 1.7 and 2.0 mm from bregma) corresponding to the ROI (within the 400 μm from the tip of the targeted sites) were chosen from each mouse, and the counting of EYFP- and/or Egr1-positive and DAPI-positive cells in the ROI was performed by an observer blind to the experimental conditions.

### Slice recordings

Details of slice recording method are described in the Supplementary Methods.

### Statistical analysis

Statistical analyses were performed using Excel (Microsoft) with StatCell 3 (OMS, Japan) and GraphPad Prism 6 (GraphPad Software, Inc., USA). Comparisons of data between two groups were analysed with Student's *t*-test (two-tailed) and multiple-group comparisons were assessed using a one-way or two-way ANOVA and followed by *post hoc* Tukey–Kramer multiple comparisons test when significant main effects were detected. Quantitative data are expressed as the mean±s.e.m.

### Data availability

The data that support the findings of this study are available from the corresponding author upon request. Detailed data for the cell count results can be seen in Supplementary Data 1.

## Additional information

**How to cite this article:** Nomoto, M. *et al*. Cellular tagging as a neural network mechanism for behavioural tagging. *Nat. Commun.* 7:12319 doi: 10.1038/ncomms12319 (2016).

## Supplementary Material

Supplementary Figures, Methods and References. Supplementary Figures 1-7, Supplementary Methods and Supplementary References.

Supplementary Data 1 Actual cell count data set. The cell count data contain actual numbers of labelled cells in Figures 3, 4, 5, 6, Supplementary Figure 4 and Supplementary Figure 6.
