# Supplementary material for: Cellular tagging as a neural network mechanism for behavioural tagging
Source: Nat Commun. 2016 Aug 1;7:12319. doi: 10.1038/ncomms12319 (PMC4974651; doi:10.1038/ncomms12319)
Supplement: Supplementary Figures, Methods and References. — Supplementary Figures 1-7, Supplementary Methods and Supplementary References. [file ncomms12319-s1.pdf]

Supplementary Figure 1.

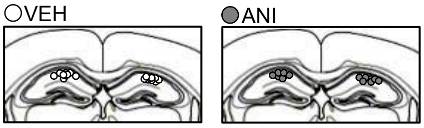

Supplementary Figure 1. Cannula tip placement in mice infused with vehicle (VEH) or anisomycin (ANI).
Cannula tip placement in mice infused with VEH (open circles) or ANI (solid circles).

Supplementary Figure 2.

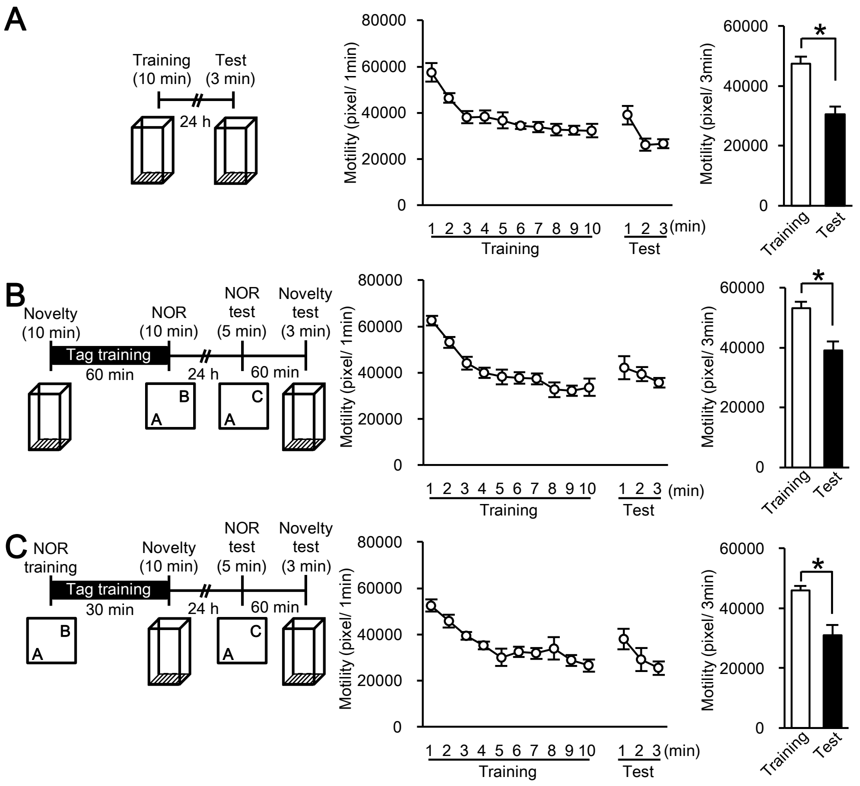

Supplementary Figure 2. Novel context exploration (NCE) training in a narrow time window leads to NOR–LTM formation.

(**A**) Novel context exploration (NCE) for place LTM. Left, NCE scheme. Middle, exploration behavior expressed as motility during training trial and test session. Right, comparison of motility in the first 3 min during the training and test sessions (n = 6; paired t test, t_5 = 4.613, p = 0.005). Data are presented as mean ± SEM. (**B**) Place LTM formed by training for 10 min followed by NOR training at an interval of 60 min. Left, behavioral paradigm. Middle, motility during training trial and test session. Right, comparison of motility in the first 3 min between training and test sessions (n = 5; paired t test, t_4 = 6.840, p = 0.002). These data were collected 60 min after the NOR test shown in **Fig. 1f**. (**C**) Place LTM formed by training for 10 min preceded by NOR training at an interval of 30 min. Left, behavioral paradigm. Middle, motility during training trial and test session. Right, comparison of motility in the first 3 min between training and test sessions (n = 6; paired t test, t_5 = 3.905, p = 0.011). These data were collected 60 min after the NOR test shown in **Fig. 1f**. Asterisk indicate a significant difference. Data are presented as mean ± SEM. Error bars indicate SEM. n, number of animals.

Supplementary Figure 3.

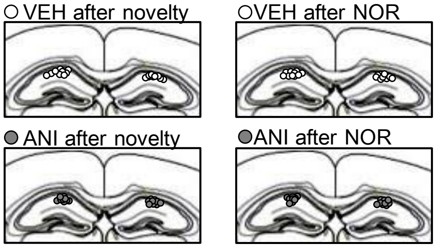

Supplementary Figure 3. Cannula tip placement in mice infused with VEH or ANI after NCE or NOR training.
Cannula tip placement in mice infused with VEH (open circle) or ANI (solid circle) after NCE (left panel) or NOR (right panel) training.

Supplementary Figure 4.

A

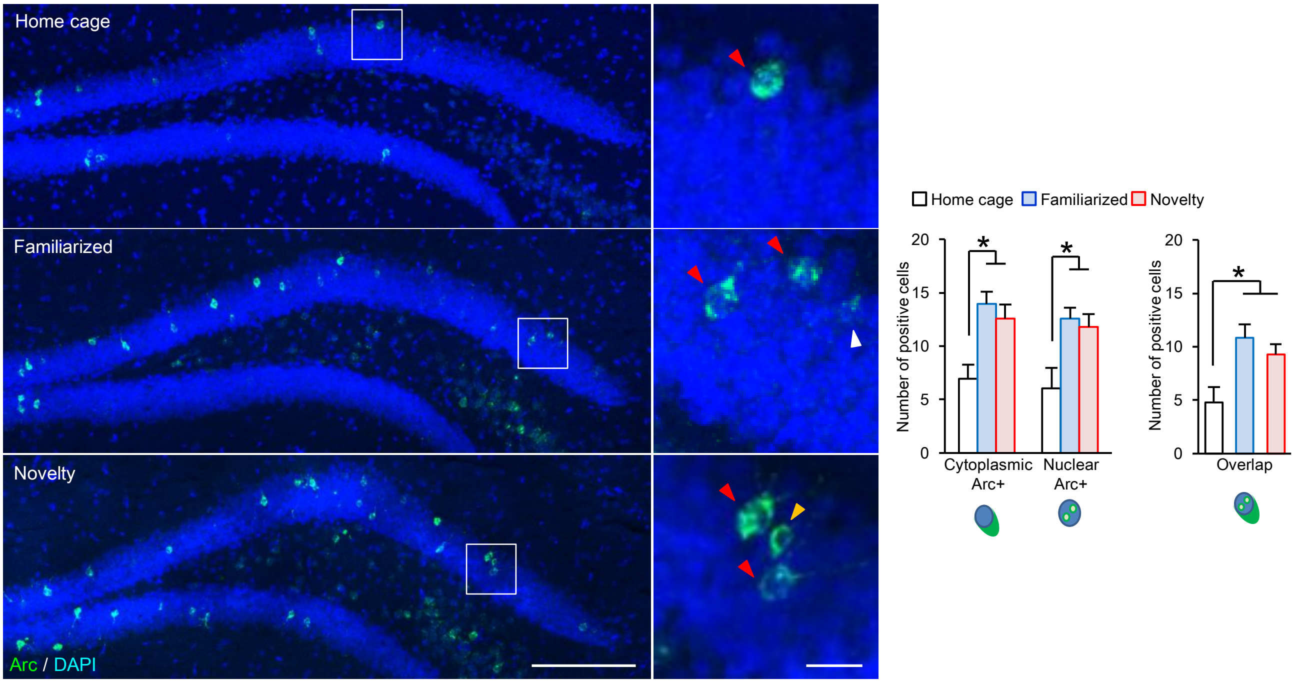

B Venn diagrams of DG results

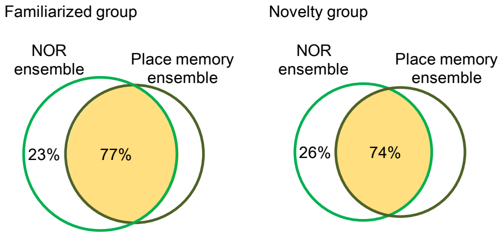

Supplementary Figure 4. Cell ensemble analyses in the dentate gyrus (DG) after behavioral tag-training.
(**A**) Detection of cells activated during behavioral tag-training. The left panels show representative z-stacks (low- and high-magnified images) of the DG region and depict DAPI-positive cells (red arrowheads) showing cytoplasmic- (yellow arrowhead) or nuclear- (white arrowhead) positive and cytoplasmic- and nuclear double-positive *Arc* RNA. Scale bar, 200 μm (left); 25 μm (right). The middle graph shows the number of DAPI-positive cells containing cytoplasmic or nuclear *Arc* RNA. The right hand graph shows the number of cytoplasmic and nuclear *Arc* RNA double-positive cells. The familiarized and novelty groups showed higher numbers of cells with cytoplasmic *Arc*, nuclear *Arc*, or double-positive *Arc* than the home cage group; the numbers in the familiarized and novelty groups were comparable (home cage, n = 6; familiarized, n = 8; novelty, n = 9; one-way ANOVA: DG cytoplasmic, $F_{2,22} = 7.183$, $p = 0.004$; DG nuclear, $F_{2,22} = 6.224$, $p = 0.007$ (with Tukey-Kramer post hoc tests); * $p < 0.05$). n, number of animals. Three sections per animal were analyzed. Data are expressed as the mean ± SEM. Error bars indicate the SEM. (**B**) Venn diagram showing the percentage overlap between NOR and NCE (place memory ensemble) in the DG. The size of each circle reflects the cell number.

Supplementary Figure 5.

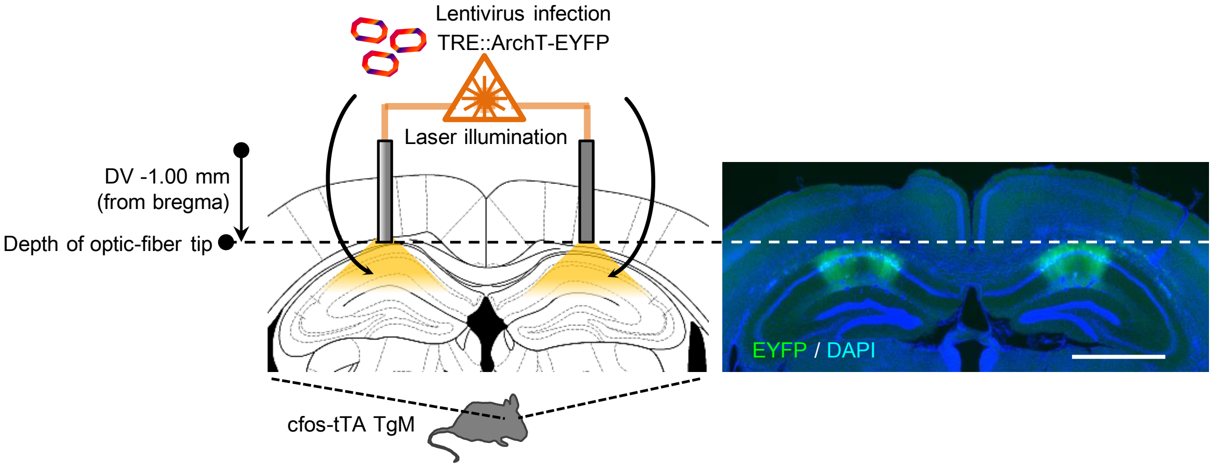

Supplementary Figure 5. Schematic illustration showing the optic fiber coordinate. An example photomicrograph showing a coronal section of the brain is also shown. c-fos::tTA mice were treated with a lentivirus (LV) harboring the TRE::ArchT–EYFP gene. The horizontal dashed line indicates the depth of the optic fiber tip coordinates from mouse bregma; the tip was targeted slightly above the hippocampi. The EYFP (green) and DAPI (blue) signals were visualized by immunostaining for EYFP, followed by fluorescence microscopy. Scale bar, 1 mm.

Supplementary Figure 6.

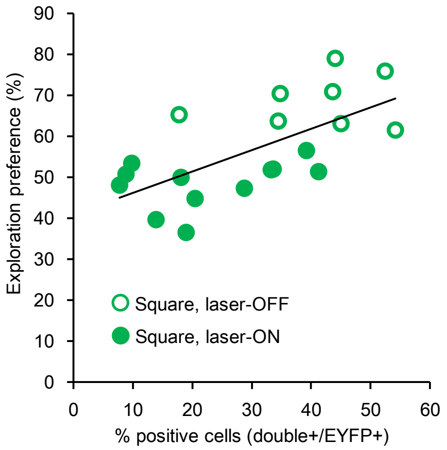

Supplementary Figure 6. Correlation analysis between the overlapping cell percentage and memory performance in the square-labeled group containing laser-OFF and –ON groups. The ratio of overlapping was correlated with the novel object preference in test session in the square-labeled group containing laser-OFF and –ON groups. Each data point shows a result obtained from one mouse (Fig. 4, Supplementary Data 1).

Supplementary Figure 7.

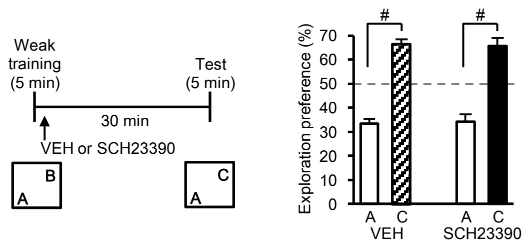

Supplementary Figure 7. Blockade of dopamine D1/D5 receptors had no effect on NOR–STM. Left, behavioral experimental scheme. The dopamine D1/D5 receptor antagonist, SCH23390, was intraperitoneally injected into the mice immediately after the 5 min period of weak NOR training and was followed by a NOR test after 30 min. Right, effect of SCH23390 on NOR–STM. Exploration preferences for familiar [A] and novel [C] objects in a test session (VEH, n = 6, t test $t_{10} = -12.56$, $p = 0.0000001$; SCH23390, n = 7, t test $t_{12} = -7.114$, $p = 0.00001$; t test for exploration preference for C between VEH and SCH23390, $t_{11} = 0.186$, $p = 0.855$). There was no significant difference between any of the groups in terms of total exploration time during training (t test $t_{11} = -0.706$, $p = 0.494$). Data are expressed as the mean ± SEM percentage of time spent exploring a particular object. #, significant difference between familiar and novel objects in each group. Error bars indicate the SEM.

Supplementary Methods

Slice recordings.

Coronal brain slices 300 µm thick from c-fos::tTA mice injected with TRE3G::ArchT 3.0-EYFP LV were prepared according to the methods described in our previous paper[1] at 24-26 h after exposure to square-type chamber. The mouse was decapitated under isoflurane anesthesia (5% in 100% O2) and the brain was rapidly removed. The block of the forebrain containing the hippocampus was dissected and cut at the midline in the ice-cold cutting solution composed of (in mM) 2.5 KCl, 0.5 CaCl2, 10 MgSO4, 1.25 NaH2PO4, 2 thiourea, 3 sodium pyruvate, 92 N-methyl-D-glucamine, 20 HEPES, 12 N-acetyl-L-cysteine, 25 D-glucose, 5 L-ascorbic acid, and 30 NaHCO3 equilibrated with 95% O2 + 5% CO2 (pH, approximately 7.4; osmolality, approximately 280 mOsm/kg). The dissected hemispheres were secured on the cutting stage of a vibrating blade slicer (VT1200S, Leica) with the rostral end upward. Coronal brain slices were prepared, incubated in a holding chamber in the cutting solution at 34°C for 15 to 20 min at first and then kept at room temperature (20–25°C) in standard artificial cerebrospinal fluid (ACSF) composed of (in mM) 125 NaCl, 3 KCl, 2 CaCl2, 1.3 MgCl2, 1.25 NaH2PO4, 10 D-glucose, 0.4 L-ascorbic acid, and 25 NaHCO3 (pH, 7.4 bubbled with 95% O2 + 5% CO2; osmolality, approximately 310 mOsm/kg) until being used in electrophysiological recordings. Following a minimum recovery time of 60 min, each slice was transferred to a recording chamber (approximately 0.4 ml volume) and fixed with nylon grids to a platinum frame. The slice was submerged in and continuously superfused at a rate of 2 ml/min with standard ACSF.

Neurons in the CA1 region were visually identified under an upright microscope (BX-51WI, Olympus) with oblique illumination. Epifluorescence images of EYFP were

captured using a CCD camera (IR-1000, DAGE-MTI) and stored digitally on a computer. Whole cell membrane potentials were recorded from visually identified EYFP-expressing neurons in the CA1. Visualization of neurons using oblique illumination allowed the selection of cells with a healthy appearance, although most neurons looked healthy. Patch-clamp pipettes made from borosilicate glass pipettes (1B120F-4; World Precision Instruments) were filled with an internal solution composed of (in mM) 120 potassium gluconate, 6 NaCl, 1 CaCl2, 2 MgCl2, 2 ATP Mg, 0.5 GTP Na, 12 phosphocreatine Na2, 5 EGTA, and 10 HEPES hemisodium (pH, 7.2 adjusted with KOH; osmolality, approximately 310 mOsm/kg). The tip resistance of the electrode was 3–8 Mohm. To stabilize the intracellular milieu, a period of 10–15 min was allowed to elapse between the giga-seal membrane rupture and the electrophysiological recordings. The membrane potential was recorded in current-clamp mode. The ArchT3.0 proteins were activated using a high-power LED illumination system (565 nm; 3-4 mW (mm)-2; Mounted High-Power LED (M565L3), Thorlabs Japan Inc., Japan) controlled by a Master-8 pulse generator (A.M.P. Instruments). We injected current to -60 mV and applied 2 sec-current steps. Light pulse duration was 1 or 2 sec. The inter-train interval was 10 sec. The membrane potential was recorded using an Axopatch 700B amplifier (Molecular Devices), filtered at 2 kHz, and digitized at 10 kHz with a 16-bit resolution using a PowerLab interface (AD Instruments). The data derived from neurons showing a resting membrane potential above -60 mV or an input resistance lower than 120 Megohms at the beginning of whole cell recording were discarded to ensure neuron vitality. The oblique illumination images and epifluorescence images were captured using the same camera without changing the recording stage and focus, and the digitally captured images were overlaid using

Photoshop software (ver. 5.5) with modifications of only the brightness and contrast. All recordings were made at room temperature (20–25°C). All compounds were purchased from Nacalai Tesque or Sigma.

Supplementary References

1. Ohkawa, N., *et al.* Artificial association of pre-stored information to generate a qualitatively new memory. *Cell Rep* **11**, 261-269 (2015).
